# Formyl-Peptide Receptor 2 Signaling Modulates SLC7A11/xCT Expression and Activity in Tumor Cells

**DOI:** 10.3390/antiox13050552

**Published:** 2024-04-30

**Authors:** Tiziana Pecchillo Cimmino, Carolina Punziano, Iolanda Panico, Zeudi Petrone, Myrhiam Cassese, Raffaella Faraonio, Vincenza Barresi, Gabriella Esposito, Rosario Ammendola, Fabio Cattaneo

**Affiliations:** 1 Department of Molecular Medicine and Medical Biotechnology, University of Naples Federico II, 80131 Naples, Italy; tiziana.pecchillocimmino@unina.it (T.P.C.); carolina.punziano@unina.it (C.P.); io.panico@studenti.unina.it (I.P.); z.petrone@studenti.unina.it (Z.P.); myrhiam.cassese@unina.it (M.C.); raffaella.faraonio@unina.it (R.F.); gabriella.esposito@unina.it (G.E.); rosario.ammendola@unina.it (R.A.); 2 Department of Biomedical and Biotechnological Sciences, University of Catania, 95123 Catania, Italy; vincenza.barresi@unict.it

**Keywords:** formyl-peptide receptor 2, NADPH oxidase, SLC7A11/xCT, glutathione, lipid peroxidation, NRF2

## Abstract

Cancer cells exhibit high levels of oxidative stress and consequently require a high amount of cysteine for glutathione synthesis. Solute Carrier Family 7 Member 11 (SLC7A11), or xCT, mediates the cellular uptake of cystine in exchange for intracellular glutamate; imported extracellular cystine is reduced to cysteine in the cytosol through a NADPH-consuming reduction reaction. SLC7A11/xCT expression is under the control of stress-inducing conditions and of several transcription factors, such as NRF2 and ATF4. Formyl-peptide receptor 2 (FPR2) belongs to the FPR family, which transduces chemotactic signals mediating either inflammatory or anti-inflammatory responses according to the nature of its ligands and/or FPR2 binding with other FPR isoforms. The repertoire of FPR2 agonists with anti-inflammatory activities comprises WKYMVm peptide and Annexin A1 (ANXA1), and the downstream effects of the intracellular signaling cascades triggered by FPR2 include NADPH oxidase (NOX)-dependent generation of reactive oxygen species. Herein, we demonstrate that stimulation of CaLu-6 cells with either WKYMVm or ANXA1: (i) induces the redox-regulated activation of SLC7A11/xCT; (ii) promotes the synthesis of glutathione; (iii) prevents lipid peroxidation; and (iv) favors NRF2 nuclear translocation and activation. In conclusion, our overall results demonstrate that FPR2 agonists and NOX modulate SLC7A11/xCT expression and activity, thereby identifying a novel regulative pathway of the cystine/glutamate antiport that represents a new potential therapeutical target for the treatment of human cancers.

## 1. Introduction

Due to their impaired metabolism, cancer cells exhibit enhanced demand and consumption of amino acids [1]. Particularly relevant in tumor cells is the requirement for cysteine, an amino acid essential not only in protein synthesis but also in the biosynthesis of reduced glutathione (GSH), which plays a key role in antioxidant defenses [2]. Cancer cells exhibit high levels of oxidative stress and, consequently, require a high demand for cysteine from the extracellular space to provide GSH synthesis [3]. However, extracellular cysteine is unstable and is quickly converted into cystine, which has an extracellular space concentration higher than cysteine [4]. Therefore, cancer cells import extracellular cystine, which is reduced to cysteine through a cytosolic NADPH-consuming reaction, which is, in turn, used for GSH synthesis.

Cancer cells show increased expression of various cell surface amino acid transporters [5], including the Solute Carrier Family 7 Member 11 (SLC7A11). SLC7A11, or xCT, is the light chain subunit of system x_c_^−^, a sodium-independent, chloride-dependent plasma membrane antiporter that mediates the cystine cellular uptake in exchange for intracellular glutamate at a molar ratio of 1:1 [6,7]. It contains 12 transmembrane domains [8], and in humans, the *SLC7A11* gene is located on chromosome 4. SLC7A11/xCT expression is under the control of stress-inducing conditions, such as hypoxia and metabolic stress, which regulate the activity of several transcription factors, including nuclear factor erythroid 2-related factor 2 (NRF2) and activating transcription factor 4 (ATF4) [9,10]. Therefore, the increased expression of SLC7A11/xCT on cancer cell surfaces enhances the uptake of cystine for intracellular GSH synthesis [11,12].

SLC7A11/xCT is primarily expressed in the brain [13] and plays functional roles in the pathophysiology of cancer and other diseases such as cardiovascular [14,15] and neurodegenerative [16] disease. Glioma cancer cells release high amounts of glutamate in the brain [17,18], suggesting the activation of the xCT system and the generation of a glutamate-rich microenvironment, which has a significant impact on the proliferation and matrix invasion of these cells [19]. In several tumors, SLC7A11 overexpression is associated with poor prognosis and drug resistance [8,20,21,22].

Formyl-peptide receptors (FPRs) are G protein-coupled receptors (GPCR) that transduce chemotactic signals and mediate either inflammatory or anti-inflammatory responses, including cell adhesion, directed migration, and NADPH oxidase (NOX)-dependent superoxide production [23]. FPR2, a member of this family, is expressed on cellular and nuclear membranes of several cell types [24,25]. The repertoire of FPR2 ligands includes non-formylated peptides, synthetic small molecules, such as WKYMVm peptide [26,27], Annexin A1 (ANXA1) with anti-inflammatory activities [28,29], and Lipoxin A4 (LXA4), a metabolite of arachidonic acid with dual anti-inflammatory and pro-resolving effects [28,30]. FPR2 can modulate pro- or anti-inflammatory responses according to the nature of its ligands and/or FPR2 binding with other FPR isoforms [31].

The activation of several kinases [32,33], phosphorylation of signaling and non-signaling proteins [26,34], and NOX-dependent generation of reactive oxygen species (ROS) [25,29,35] are part of the downstream effects of the intracellular signaling cascades triggered by FPR2. NOXs are cell membrane-associated complexes that generate ROS as their primary function [36,37]. They include seven isoforms: NOX1-5 and dual oxidase (DUOX) 1–2, which have similarities in both structure and enzyme function. NOXs are expressed in a cell- or tissue-specific manner and are activated by specific mechanisms and regulatory subunits. NOX2 is the most widely distributed NOX isoform in humans [36]. p22^phox^ is a membrane partner protein and is required for NOX2 activation [38]. Other cytosolic partner proteins include RAC1, p67^phox^/NOX activator 1 (NOXA1), and p47^phox^/NOX organizer 1 (NOXO1). The activation of NOX2 requires the phosphorylation of p47^phox^, which interacts with p22^phox^ and translocates on the membrane with other cytosolic subunits [36]. The fully assembled NOX2 complex generates O_2_^−.^ through the transfer of an electron from NADPH in the cytosol to molecular oxygen in the extracellular space [39].

FPR-mediated NOX-dependent ROS production acts as a second messenger in the molecular mechanisms responsible for tyrosine kinase receptor (TKR) transactivation and, in turn, in the activation of specific TKR-dependent intracellular signaling pathways [27,40,41,42].

Previously, in a phospho-proteomic analysis, we demonstrated that FPR2 stimulation of the human CaLu-6 epithelial carcinoma cell line induced the redox-regulated phosphorylation of several proteins involved in cellular primary metabolism [26,34]. Furthermore, by a metabolomic approach, we demonstrated that the metabolic pathways of glucose, glutamine, and pyrimidine nucleotides are activated in FPR2-stimulated CaLu-6 cells [40,43]. Herein, we analyze the metabolic fate of glutamate in WKYMVm- and ANXA1-stimulated cells and prove the redox-regulated activation of SLC7A11/xCT antiporter.

## 2. Materials and Methods

### 2.1. Cell Cultures and Reagents

Anaplastic lung cancer CaLu-6 cells and HCT 116 human colon cancer cells (ATTC, Manassas, VA, USA) were grown in Dulbecco’s modified Eagle medium (DMEM), completed with 10% fetal bovine serum (FBS) (Invitrogen Corp., Carlsbad, CA, USA) at 37 °C and 5% CO_2_ until 70% confluency and serum-starved for 24 h. Serum-deprived CaLu-6 cells were exposed or not to 10 μM WKYMVm (Primm, Milan, Italy) or 10 nM annexin A1 (ANXA1) (Bio-Techne, Minneapolis, MN, USA) at different times. In other experiments, cells were pretreated with 10 μM WRWWWW (WRW4) (Primm, Milan, Italy) for 15 min or 100 μM apocynin (Sigma Chemical, St. Louis, MO, USA) for 2 h before the incubation with WKYMVm or ANXA1.

### 2.2. p22phoxCrispr/Cas9 Double-Nickase CaLu-6 Cells

p22phoxCrispr/Cas9 cells were created as previously described [40]. CaLu-6 cells were transfected with a Double Nickase Plasmid or a negative control double Nickase Plasmid (Santa Cruz Biotechnology, Irvine, CA, USA). p22phoxCrispr/Cas9 and negative control clones were selected for puromycin and p22phox expression was tested by Western blotting [39]. p22phox knockout and p22phox negative control clones were collected to obtain p22phoxCrispr/Cas9 and negative control CaLu-6 cells.

### 2.3. Protein Extraction and Western Blot

Whole protein lysates were obtained as described elsewhere [44] by incubating the cells with ice-cold RIPA buffer (50 mM Tris–HCl, pH 7.4, 150 mM NaCl, 1% NP-40, 1 mM EDTA, 0.25% sodium deoxycholate, 1 mM NaF, 10 μM Na_3_VO_4_, 1 mM phenylmethylsulfonylfluoride, 10 μg/mL aprotinin, 10 μg/mL pepstatin, 10 μg/mL leupeptin). Nuclear protein purification was performed with a Qproteome kit (Qiagen, Hiden, Germany) following the manufacturer’s instructions.

Bradford protein assay was used to determine protein concentration (BioRAD, Hercules, CA, USA). Western blot analysis was performed as previously described [44]. Anti-xCT/SLC7A11 was from Cell Signalling Technology (Danvers, MA, USA). Anti-NRF2, anti-LAP2, anti-GAPDH, anti-tubulin were purchased from Santa Cruz Biotechnology (Irvine, CA, USA). All primary antibodies were diluted 1:1000 *v*/*v*.

The protein bands were visualized by enhanced chemiluminescence reagent (Amersham Biosciences, Little Chalfont, Buckinghamshire, UK) and quantified by densitometric analysis (Chemidoc, Bio-Rad, Hercules, CA, USA). Full blots are available in the Supplementary Materials (Supplementary Figures S2–S6).

Each experiment and densitometric quantification were separately repeated at least three times.

### 2.4. Glutathione Assay

Glutathione Assay Kit (Sigma Chemical, St. Louis, MO, USA) was used to measure total glutathione according to manufacturer’s instructions. Briefly, for each experimental point, 2 × 10^6^ cells were washed in cold phosphate buffer saline (PBS) and lysed in 200 μL of cold buffer containing 50 mM phosphate, pH 7, and 1 mM EDTA. Lysate samples were deproteinated with a 5% (by weight) meta-phosphoric acid solution (MPA Reagent) in purified water and incubated with working reagent solution. The optical density (OD) of each sample was read at 412 nm at zero and after 10 min.

### 2.5. Lipid Peroxidation Assay

Lipid peroxidation was evaluated with Image-iT^®^ Lipid peroxidation Kit (Invitrogen, Carlsbad, CA, USA) following manufactures instructions. Briefly, 2 × 10^4^ cells were plated on µ-Slide 8 wells (Ibidi GmbH, Gräfelfing, Germany), and the day after were exposed or not to 10 mM WKYMVm or 10 nM ANXA1 for 3, 6, or 12 h before the incubation for 2 h with 100 mM cumene hydroperoxide to induce lipid peroxidation. Untreated cells were used as a negative control, whereas exposure to cumene hydroperoxide was used as a positive control (PMID: 31901729). Thirty minutes before the conclusion of cell treatments, cells were incubated with 10 mM Image-iT^®^ Lipid Peroxidation Sensor, which is 581/591 nm C11 reagent and is a sensitive fluorescent report for lipid peroxidation. Nuclei staining was performed by incubating cells with Hoechst 33343 (Invitrogen, Carlsbad, CA, USA). Z-slice images were acquired with a Leica Thunder Imaging System (Leica Microsystems, Wetzlar, Germany) equipped with a LEICA DFC9000 GTC camera, Lumencor fluorescence LED light source, and 63× oil immersion objective. ImageJ software Version 1.53 was used for the quantitative fluorescence ratio analysis of green signals at 527 nm (representing peroxidized lipids)/red signals at 590 nm (representing non-peroxidized lipids).

### 2.6. Immunofluorescence

CaLu-6 cells were fixed with phosphate buffer containing 4% (*w*/*v*) paraformaldehyde for 30 min at Room Temperature (RT), then blocked and permeabilized with 5% (*w*/*v*) BSA, 0.2% (*v*/*v*) Triton X-100 (Sigma, Saint Louis, MO, USA) and 10% (*v*/*v*) FBS in PBS for 30 min at RT. Then, cells were stained with primary NRF2 antibody (1:200) (H-300 sc-130332, Santa Cruz) and anti-rabbit Alexa Fluo 488-conjugated secondary antibody (1:200) (Invitrogen, Carlsbad, CA, USA). Nuclei staining was performed by incubating cells with Hoechst 33342 (Bio-Rad Laboratories). Immunofluorescence was analyzed by Leica Thunder Imaging System (Leica Microsystems, Wetzlar, Germany) equipped with a LEICA DFC9000 GTC camera and Lumencor fluorescence LED light source. Z-slice images were acquired using 63× oil immersion objective.

### 2.7. Statistical Analysis

Data are shown as means ± SEM. Differences between groups were assessed for statistical significance using the one-way analysis of variance (ANOVA) and then determined with the least significant difference test. Data were considered statistically significant with *p* < 0.05.

## 3. Results and Discussion

### 3.1. FPR2 Agonists Induce SLC7A11/xCT Expression in CaLu-6 Cells

A metabolomic analysis performed in FPR2-stimulated CaLu-6 cells revealed enhanced concentrations of glutamine and glutamate [43]. Glutamate can derive from glutamine through a reaction catalyzed by glutaminase, and, accordingly, we previously demonstrated that the glutamine transporter ASCT2 is up-regulated in FPR2-stimulated CaLu-6 cells and that ASCT2 over-expression correlates with an increased uptake of glutamine [43]. Glutamate dehydrogenase catalyzes the NAD(P)H-dependent conversion of glutamate in α-ketoglutarate, the most important carbon source in the tricarboxylic acid cycle [45]. Therefore, glutamate represents a crucial player in cancer cells characterized by rapid proliferation and high metabolism rate [46]. Glutamate also participates in the biosynthesis of GSH, a tripeptide composed of cysteine, glycine, and γ-linked glutamate [2], which is involved in the maintenance of redox homeostasis. 

In normal cells, de novo synthesis of cysteine can cope with the GSH biosynthetic demand; on the contrary, since high levels of oxidative stress are detectable in cancer cells, biosynthesis or catabolic supply of cysteine resulting from protein degradation is insufficient to meet the demand for antioxidant defense systems [47]. 

**Figure 1:**
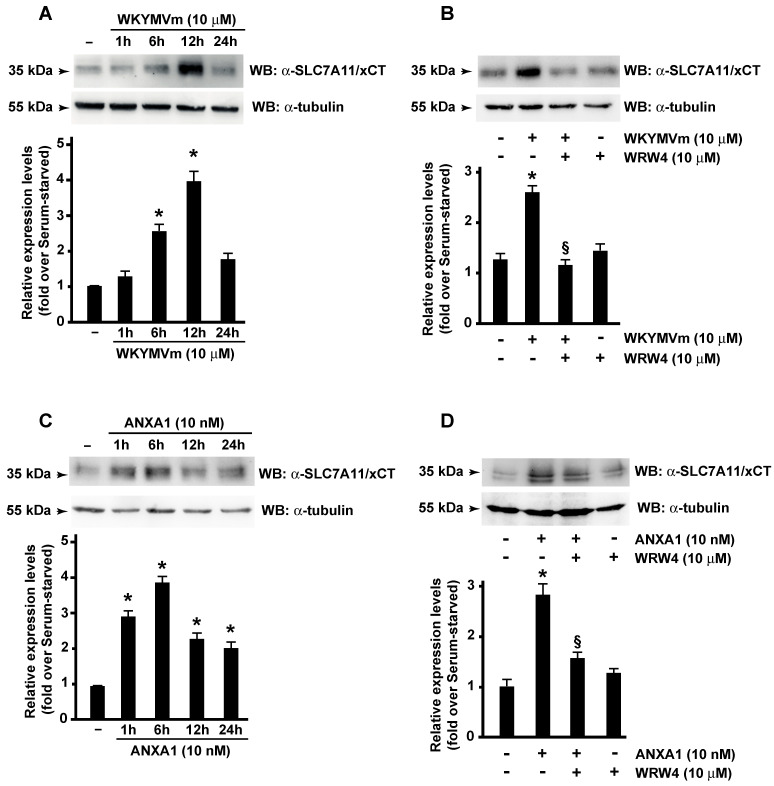
Formyl-peptide receptor 2 (FPR2) stimulation promotes SLC7A11/xCT expression. CaLu-6 cells were serum-starved for 24 h and then stimulated for 1, 6, 12, or 24 h with WKYMVm (**A**) or ANXA1 (**C**) or preincubated for 15 min with WRWWWW (WRW4) before the stimulation with WKYMVm for 12 h (**B**) or ANXA1 for 6 h (**D**). Fifty-five micrograms of whole lysates were resolved on 10% SDS-PAGE and incubated with an anti-SLC7A11/xCT (α-SLC7A11/xCT) antibody. An anti-tubulin (α-tubulin) antibody was used as a control of protein loading. Data are representative of 4 different experiments. * *p* < 0.05 compared to unstimulated cells. § *p* < 0.05 compared to stimulated cells.

Since SLC7A11/xCT represents the major regulator of metabolic reprogramming in cancer cells that depend on extracellular cystine for survival [48], we analyzed its expression in FPR2-stimulated CaLu-6 cells. We observed that both WKYMVm and ANXA1 induce a time-regulated overexpression of SLC7A11/xCT (Figure 1A,C), which was prevented by preincubation with WRWWWW peptide (WRW4), an FPR2 antagonist (Figure 1B,D). The time course was different for the two FPR2 agonists. Cells exposed to WKYMVm showed a maximum expression of SLC7A11/xCT after 12 h (Figure 1A), whereas in ANXA1-stimulated CaLu-6 cells, maximum expression of SLC7A11/xCT was detectable after 6 h (Figure 1C). The different behavior of the two agonists reflects the promiscuity of FPR2, which is activated by a large number of ligands and thus is subjected to different conformational changes upon ligand binding [49]. Consistently, we observed both WKYMVm- and ANXA1-time regulated increase of SLC7A11/xCT expression in HCT 116 human colon cancer cells (Supplementary Figure S1), which constitutively express FPR2 [50,51].

### 3.2. FPR2-Dependent SLC7A11/xCT Expression Requires a Functional NADPH Oxidase

Cancer cells aberrantly reprogram several metabolic pathways to adapt themselves to environmental changes and to support the high-energy demands, thus promoting survival and proliferation. Metabolic reprogramming rewires resources for survival and allows cancer cells to maintain a reduction–oxidation balance [52]. The improved energy metabolism that supports the enhanced proliferation rate further increases oxidative stress [53]. ROS can be beneficial at low levels in the promotion of cellular signaling and in the maintenance of cellular homeostasis [54]. In tumor cells, an enhanced production of ROS, or dysregulation of ROS levels, results in oxidative stress, which can contribute to cancer initiation and progression [55,56,57]. High levels of ROS concentration induce cell death by DNA damage. Cells developed different antioxidant defense mechanisms to neutralize ROS and thus maintain redox balance in eukaryotic cells. While in the cytosol, a reducing environment is generally preserved, the extracellular environment is highly oxidizing. SLC7A11/xCT maintains the cystine/cysteine redox cycle across the cell membrane to regulate oxidative stress in the tumor microenvironment [58]. Residual cysteine from GSH synthesis is exported and quickly oxidized to cystine. Extracellular cystine is continuously imported by SLC7A11/xCT, thus generating the cystine/cysteine redox cycle, resulting in a reduced extracellular environment that promotes the growth and survival of cancer cells [59]. Other efficient antioxidant cellular defense systems include non-thiol-dependent, such as superoxide dismutase and catalase enzymes, and thiol-dependent antioxidant systems that include the thioredoxin (Trx) system, which acts through its disulfide reductase activity [60,61,62,63].

**Figure 2:**
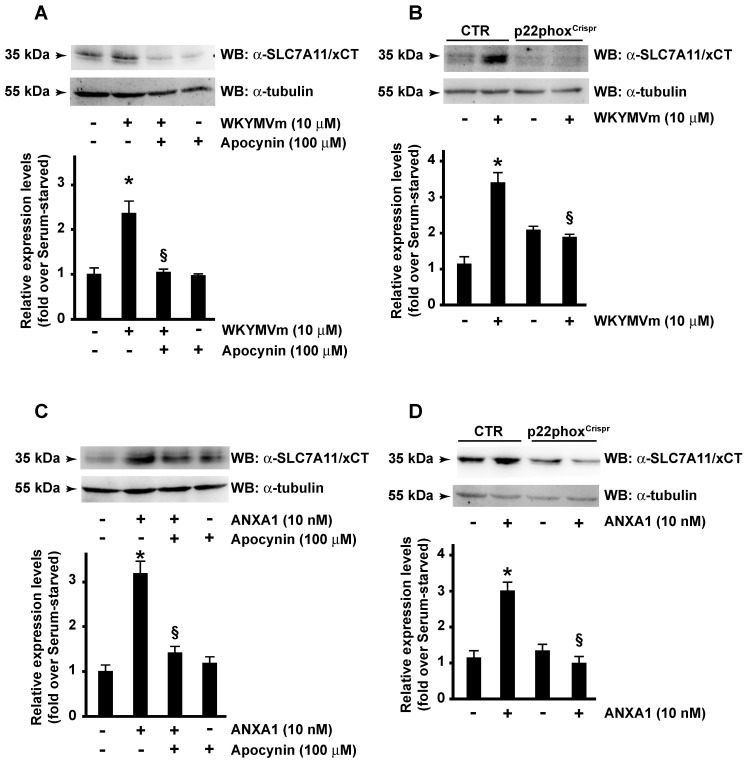
Formyl-peptide receptor 2 (FPR2)-induced SLC7A11/xCT expression depends on NADPH oxidase activity. Serum-starved CaLu-6 cells were pretreated with apocynin (**A**,**C**) and then stimulated with WKYMVm (**A**) or ANXA1 (**C**). CaLu-6-control^Crispr/Cas9^ cells (CTR) and p22phox^Crispr/Cas9^ (p22phox^Crispr^) cells were serum-starved for 24 h and then stimulated with WKYMVm (**B**) or ANXA1 (**D**). Fifty-five micrograms of whole lysates were resolved on 10% SDS-PAGE and incubated with an anti-SLC7A11/xCT (α-SLC7A11/xCT) antibody. An anti-tubulin (α-tubulin) antibody was used as a control of protein loading. Data are representative of 3 different experiments. * *p* < 0.05 compared to unstimulated cells. § *p* < 0.05 compared to stimulated cells.

Several diseases, including cancer, depend on the aberrant activation and/or expression of NOXs, which play a key role in the regulation of the metabolism of cancer [40,43,64,65]. Previously, we demonstrated that in non-phagocytic cells, FPR2 stimulation triggers ERK-, PKCα-, and PKCδ-dependent p47^phox^ phosphorylation and NOX activation [25,66]. Therefore, we analyzed the role of ROS generated by NOX in the FPR2-dependent SLC7A11/xCT overexpression. CaLu-6 cells were preincubated with apocynin, a reversible inhibitor of NOX activity that hampers the assembly of the p47^phox^ subunit with the membrane complex [67], and then stimulated with the FPR2 agonists. Obtained results show that either WKYMVm- or ANXA1-dependent SLC7A11/xCT expression was prevented by blocking NOX functions (Figure 2A,C). By CRISPR/Cas9-based genome editing, we generated a Calu-6 cell line expressing a non-functional form of p22^phox^, a membrane partner of NOX (p22phox^Crispr/Cas9^) [29] and, consistently, we observed that in these cells, the ability of WKYMVm and ANXA1 to induce SLC7A11/xCT expression was prevented (Figure 2B,D).

Taken together, these results prove that FPR2-dependent SLC7A11/xCT expression requires a functional NADPH oxidase.

### 3.3. WKYMVm and ANXA1 Stimulation Induces the Synthesis of Glutathione

GSH and Trx systems depend on NADPH for their reducing power. Interestingly, NADPH is also the substrate of NOXs and a regulator of their enzyme activity [68]. NADPH derives from the metabolism of glucose through the pentose phosphate pathway (PPP), where glucose-6-phosphate dehydrogenase represents the rate-limiting enzyme [69]. Previously, we demonstrated that FPR2 stimulation by WKYMVm or ANXA1 promotes NADPH production and activates the non-oxidative phase of PPP [43].

SLC7A11/xCT imports extracellular cystine in exchange for intracellular glutamate. Cysteine is further utilized to synthesize GSH through a two-step process that first produces γ-glutamylcysteine from cysteine and glutamate by γ-glutamylcysteine synthetase, then GSH from γ-glutamylcysteine by glutathione synthetase [8,70,71]. We previously demonstrated that ASCT2 transporter expression is up-regulated in FPR2-stimulated cells and is correlated to an increased uptake of glutamine [43]. Therefore, glutamate needed for GSH synthesis can be derived by glutamine through a reaction catalyzed by glutaminase.

**Figure 3:**
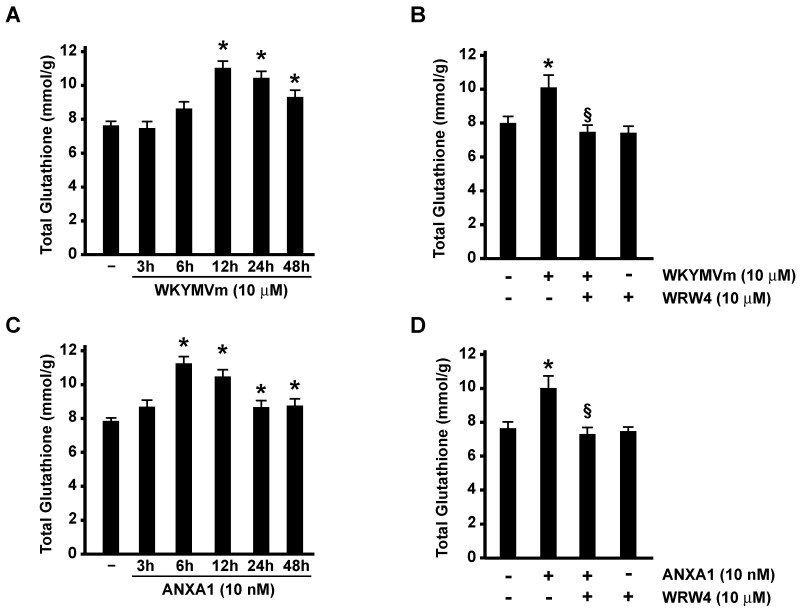
WKYMVm and ANXA1 promote an increase in total glutathione levels. Serum-starved CaLu-6 cells were stimulated with WKYMVm (**A**) or ANXA1 (**C**), and total glutathione was measured according to manufacturer’s instruction. Cells were also preincubated for 15 min with WRWWWW (WRW4) and then stimulated with WKYMVm (**B**) or ANXA1 (**D**). Data are representative of 3 different experiments. * *p* < 0.05 compared to unstimulated cells. § *p* < 0.05 compared to stimulated cells.

We measured total glutathione in FPR2-stimulated cells and observed that both WKYMVm and ANXA1 induce a time-dependent increase of glutathione concentration (Figure 3A,C), which was prevented by the FPR2 antagonist WRW4 (Figure 3B,D). In WKYMVm- and ANXA1-stimulated cells, the maximum glutathione concentration is detectable after 12 h and 6 h, respectively (Figure 3A,C) and correlates with the maximum expression of SLC7A11/xCT induced by the two agonists (Figure 1A,C). FPR2-dependent enhanced concentration of glutathione strictly depends on receptor stimulation since preincubation with WRW4 prevents glutathione synthesis (Figure 3B,D).

### 3.4. Lipid Peroxidation Is Prevented in FPR2-Stimulated CaLu-6 Cells

Membrane polyunsaturated fatty acid phospholipids (PUFA-PLs) are susceptible to peroxidation due to the presence of weak C-H bonds [72]. Non-enzymatic lipid peroxidation starts when an ·OH mediates the remotion of a bisallylic hydrogen atom from PUFA-PLs, resulting in the production of a PL radical (PL·). The peroxyl radical (PLOO·) is generated when PL· reacts with molecular oxygen. PLOO· then removes hydrogen from another PUFA and creates PL hydroperoxides (PLOOH). PLOOH production is propagated by lipid free radicals, primarily PLOO· and alkoxyl PL radicals (PLO·), reacting with PUFA-PLs [73]. This process results in the accumulation of dangerous lipid peroxides, which have an impact on the disruption of plasma membrane integrity and organelle membranes [74]. 

In the enzymatical lipid peroxidation process, several nonheme iron dioxygenases, such as lipoxygenases (LOXs), prostaglandin synthase 2/cyclooxygenase 2, and cytochrome P450 reductase, oxidize PUFA-PLs into PLOOH through their catalytic activities [75]. Long-chain saturated fatty acids (SFA) are another target of lipid peroxidation. The peroxisomal enzyme fatty acyl-CoA reductase 1 reduces SFA to fatty alcohols, which are incorporated into the sn-1 position of PUFA-PLs, generating polyunsaturated ether phospholipids which can be hyperoxidized [14,76].

PUFA oxidation increases when GSH in cells is exhausted [77], proving that it strictly depends on GSH concentration. Since SLC7A11/xCT antiporter provides cysteine for GSH synthesis, its inactivation potently induces lipid peroxidation, and conversely, its overexpression in cancer cells promotes GSH biosynthesis and prevents lipid peroxidation [4].

**Figure 4:**
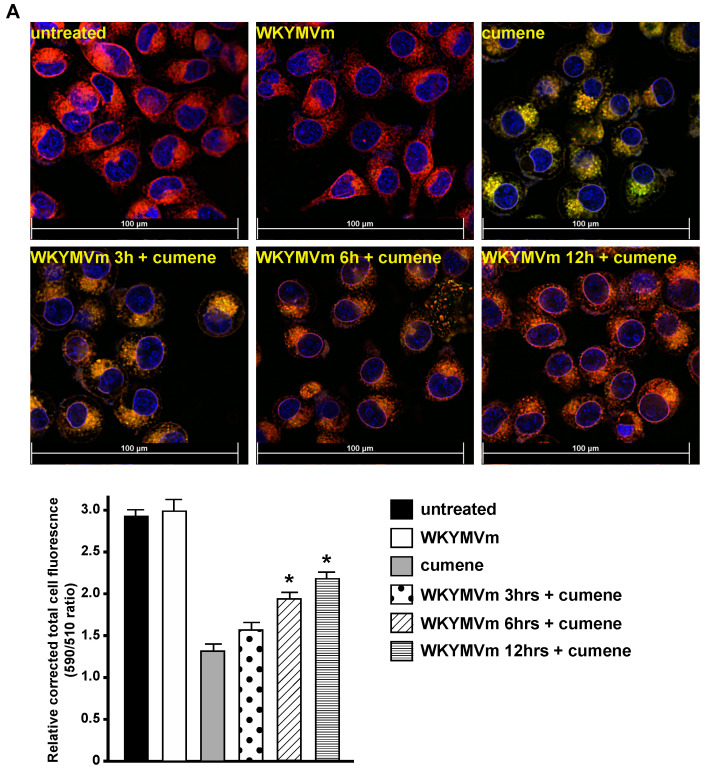

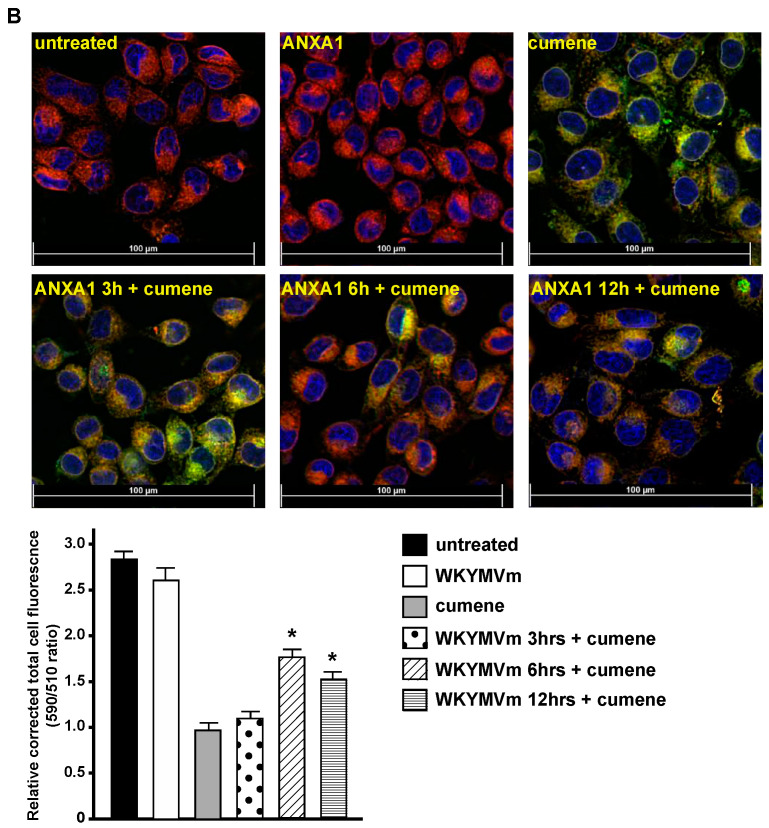
Incubation with WKYMVm or ANXA1 protects CaLu-6 cells from lipid peroxidation. Serum-starved CaLu-6 cells (untreated) were exposed for 3, 6, or 12 h (hrs) to WKYMVm (**A**) or ANXA1 (**B**) before the incubation for 2 h with 100 μM cumene hydroperoxide (cumene). Cells were stained with BODIPY 581/591 C11 fluorescent reporter for lipid peroxidation; the reagent shifted the fluorescence emission peak from 590 nm (red) to 510 nm (green). Nuclei of live cells were stained with Hoechst 33342 (blue). Quantitative fluorescence analysis is reported as the ratio of red signal at 590 nm (representing non-peroxidized lipids)/green signal at 510 nm (representing peroxidized lipids). Figures show representative fluorescence merged images of lipid peroxidation. Scale bar = 100 μm. All data are representative of three independent experiments. * *p* < 0.05 compared to cumene hydroperoxide (cumene) treated cells.

Since we found that, in CaLu-6 cells, FPR2 stimulation with two different agonists induced SLC7A11/xCT overexpression (Figure 1) and increased glutathione concentration (Figure 3), we also analyzed lipid peroxidation and showed that either WKYMVm or ANXA1 prevented time-dependent lipid peroxidation induced by cumene hydroperoxide (Figure 4A,B).

### 3.5. Nuclear Translocation of NRF2 Depends on FPR2 Stimulation

SLC7A11/xCT expression can be modulated by hypoxia, amino acid starvation, metabolic stress, genotoxic stress, and other stress-inducing conditions, but the main molecular mechanism of control is transcriptional regulation. NRF2 and ATF4 are two transcription factors that regulate stress-induced SLC7A11/xCT transcription [78,79]. Kelch-like ECH-associated protein (KEAP1) acts as a cytoplasmic repressor of the NRF2 oxidative responsive transcription factor. Under normal physiological conditions, NRF2 is inhibited by binding to KEAP1 and degraded in the proteasome. In cellular stress conditions, the proteasomal degradation of NRF2 is hampered, leading to its stabilization and nuclear translocation. In the nucleus, NRF2 heterodimerizes with an array of transcriptional regulatory proteins, such as Jun and Fos, and interacts with antioxidant response cis-elements (AREs) located within promoter regions regulating SLC7A11/xCT transcription [80]. NRF2 overexpression enhances SLC7A11/xCT levels, resulting in increased GSH biosynthesis and in the protection of cells from oxidative damage [16]. The transcription of several other antioxidant response-associated target genes, such as NADPH-generating enzyme genes, is also regulated by NRF2 [81].

ATF4 binds to cis-response elements located in promoter regions of the SLC7A11/xCT gene, allowing the cells to adapt and respond to stress [82]. NRF2 and ATF4 can interact and regulate SLC7A11/xCT expression cooperatively [82]. In addition to the transcriptional regulation, SLC7A11/xCT is also regulated through post-transcriptional or post-translational mechanisms, protein–protein interactions, and transporter activity [8,83].

**Figure 5:**
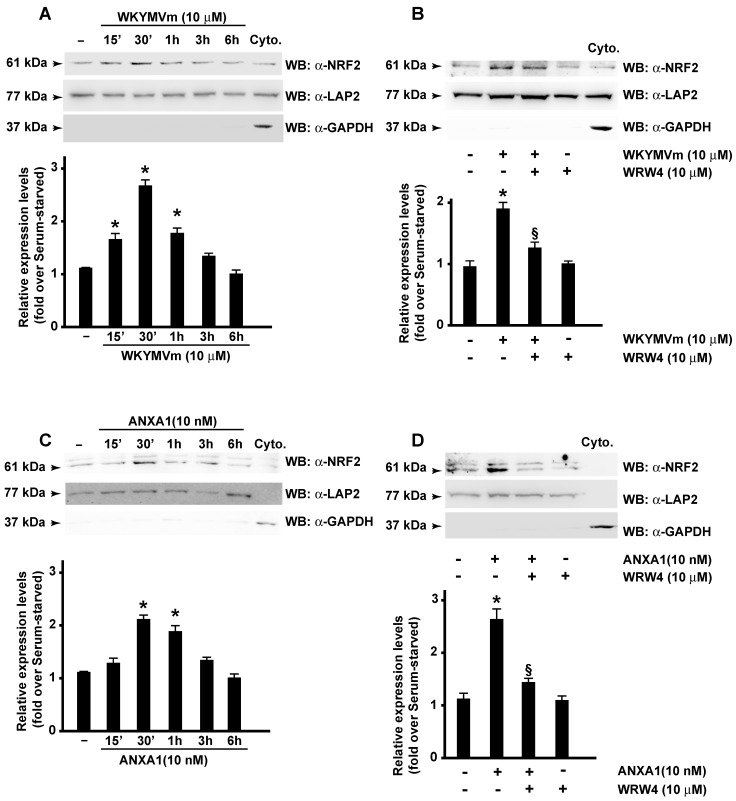
Formyl-peptide receptor 2 (FPR2) stimulation promotes NRF2 nuclear translocation. Serum-starved CaLu-6 cells were stimulated for the indicated times with WKYMVm (**A**) or ANXA1 (**C**) in the presence or absence of WRWWWW (WRW4) (**B**,**D**). Fifty micrograms of nuclear lysates were resolved on 10% SDS-PAGE and probed with an anti-NRF2 antibody (α-NRF2). An anti-LAP2 (α-LAP2) antibody was used as a control of protein loading. Fifty micrograms of a cytosolic fraction (Cyto.) was loaded, and an anti-GAPDH antibody (α-GAPDH) was used as a control of cytosolic proteins. Data are representative of 3 different experiments. * *p* < 0.05 compared to unstimulated cells. § *p* < 0.05 compared to stimulated cells.

**Figure 6:**
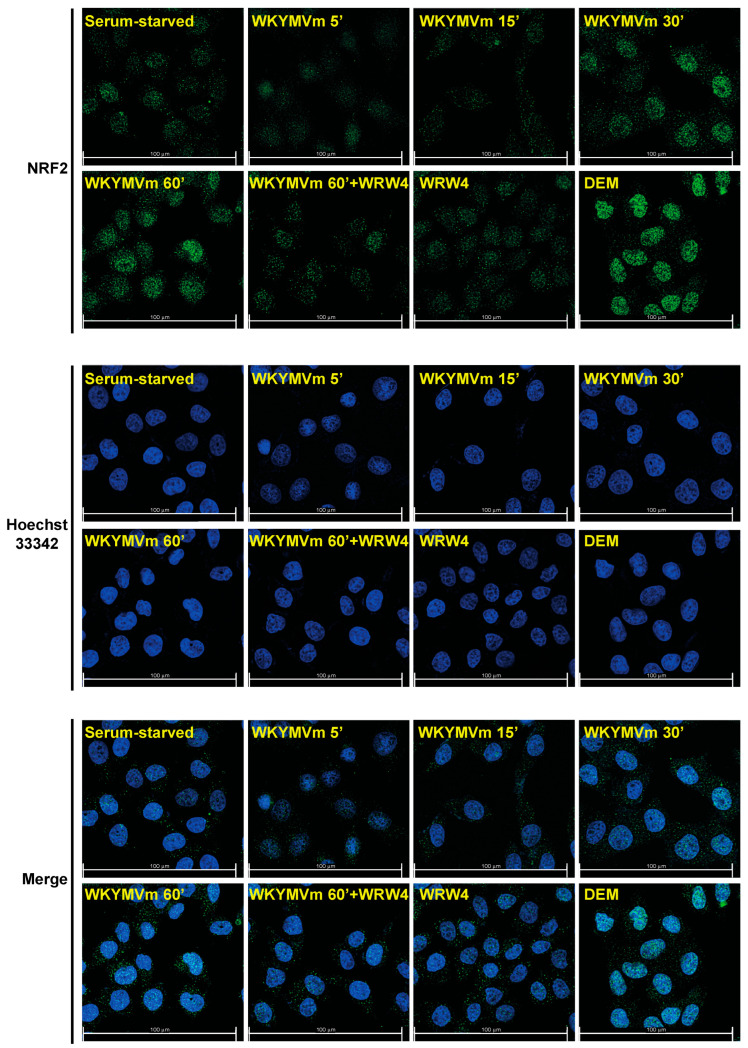
WKYMVm promotes formyl-peptide receptor 2 (FPR2)-dependent NRF2 nuclear translocation. Nuclear localization of NRF2 in WKYMVm-stimulated cells was detected by using immunofluorescence staining. Cells were serum-deprived for 24 h and then incubated with WKYMVm for the indicated times in the presence or absence of an FPR2 antagonist. Incubation for 2 h with 200 mM diethyl maleate (DEM) was used as a positive control of NRF2 nuclear translocation. Cells were incubated with an anti-NRF2 primary antibody followed by incubation with an anti-rabbit Alexa Fluo 488-conjugated secondary antibody (NRF2, green signal). To visualize vital nuclei, cells were also incubated with Hoechst 33342 (blue signal). Images were captured and merged using the Leica Thunder Imaging System (merge). Data are representative of 4 different experiments.

**Figure 7:**
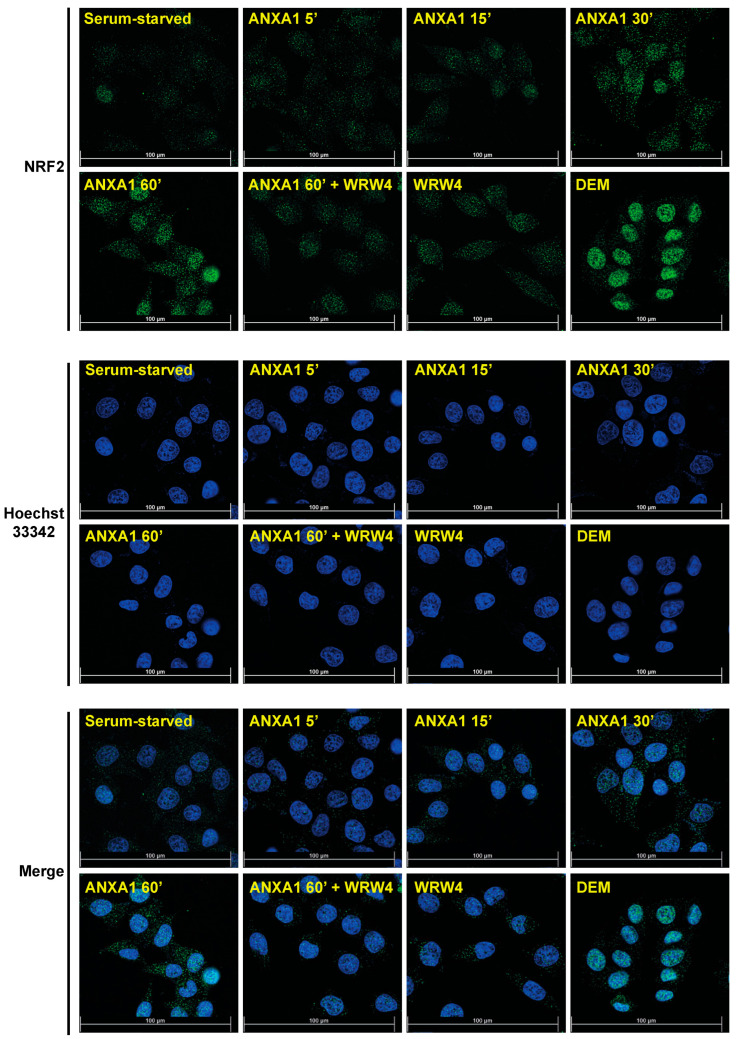
Annexin A1 (ANXA1) enhances NRF2 nuclear translocation in a formyl-peptide receptor 2 (FPR2)-dependent manner. Immunofluorescence staining of CaLu-6 cells exposed to ANXA1. Serum-starved cells were stimulated with ANXA1 for the indicated times in the presence or absence of WRW4. Incubation for 2 h with 200 mM diethyl maleate (DEM) was used as a positive control of NRF2 nuclear translocation. Cells were incubated with an anti-NRF2 primary antibody followed by incubation with an anti-rabbit Alexa Fluo 488-conjugated secondary antibody (NRF2, green signal). To visualize vital nuclei, cells were also incubated with Hoechst 33342 (blue signal). Images were captured and merged using the Leica Thunder Imaging System (merge). Data are representative of 4 different experiments.

In Western blot experiments, we observed a time-dependent increase of nuclear NRF2 in either WKYMVm- or ANXA1-stimulated CaLu-6 cells (Figure 5A,C). Furthermore, the preincubation WRW4 prevents NRF2 nuclear translocation (Figure 5B,D). Consistently, also immunofluorescence assays revealed FPR2-dependent NRF2 nuclear translocation upon WKYMVm (Figure 6) and ANXA1 (Figure 7) stimulation. Similarly, in ARPE-19 cells, LXA4, which exerts its anti-inflammatory, pro-resolving, and antioxidant effects by binding to FPR2 [84], ameliorates NRF2 nuclear translocation and its DNA-binding activity [85]. Resolvin D1 (RvD1) also promotes inflammation resolution by binding to FPR2 [86], but its effects on NRF2 and NOX-dependent ROS generation are different. RvD1-mediated protection is mediated by inhibition of assembly of the NOX2 complex via prevention of p47^phox^ phosphorylation, lipid peroxidation, and NRF2 translocation [87].

## 4. Conclusions

The process of tumorigenesis is largely driven by metabolic reprogramming. A significant alteration in metabolic pathways leads to metabolic rewiring and upregulation of the production of ROS in tumor cells. Therefore, understanding the mechanisms behind these metabolic changes and ROS production may hold crucial implications for the development of effective cancer therapies. Cellular stress conditions are responsible for NRF2 heterodimerization and its interaction with ARE cis-elements. The observation reported in this study, that FPR2 signaling triggers NRF2 activation, leads to the question of how many and which genes are NRF2-regulated upon FPR2 stimulation. The identification of these genes could reflect new potential targets for therapies for human cancers. In this study, we have not focused on FPR2-dependent NRF2-regulated genes, and further studies are required for their identification. Oxidative stress is also responsible for the accumulation of peroxided lipids, which are involved in ferroptosis processes. Ferroptosis is iron-dependent cell death, different from apoptosis, necrosis, autophagy, and other forms of cell death [88]. Our results show that FPR2 signaling prevents lipid peroxidation. However, we did not investigate ferroptosis processes in FPR2-stimulated CaLu-6 cells.

Cancer metabolism is characterized by the deregulated uptake of amino acids that arises from a complex interplay between signaling pathways and transporters compared to adjacent normal tissue tumors showing high expression of several members of the amino acid transporters family, which is associated with tumor progression, clinical outcome, and treatment resistance. While significant attention has been given to the functional characterization of amino acid transporters in various human malignancies, research on their potential use as molecular biomarkers and therapeutic targets is still in its early stages. Therapeutic targeting of amino acid transporters is difficult since tumors often activate survival mechanisms, such as autophagy, when amino acid levels are low to overcome nutrient stress. However, inhibiting amino acid uptake can enhance cell death and sensitivity to conventional cancer treatments, especially in autophagy-deficient tumor cells. Although several preclinical methods could potentially inhibit amino acid transporters, none of them have been tested in clinical trials yet.

SLC7A11/xCT is highly expressed in a variety of tumors, and this correlates with the proliferation, invasion, metastasis, and drug resistance of cancer. This antiporter is an attractive target for anti-cancer therapy due to its role in GSH synthesis. Although the targeted therapy of tumors with SLC7A11/xCT has been investigated [22,52], many observations indicate that inhibitors of this antiport lack specificity and that several associated side effects may limit their functions. Consequently, the identification of extremely successful and specific SLC7A11/xCT inhibitors is of great relief for the development of new drugs for the treatment of tumors. In this context, the observation that FPR2 agonists and NOX modulate SLC7A11/xCT expression and activity provides alternative possibilities to control the cystine/glutamate antiport and thus to develop new therapeutical strategies for the treatment of human cancers.

## Data Availability

The data presented in this study are available within the article. Other data that support the findings of this study are available upon request to the corresponding authors.

## Supplementary Materials

The following supporting information can be downloaded at: https://www.mdpi.com/article/10.3390/antiox13050552/s1, Figure S1: Supplementary Figure S1; Figure S2: Full Blot x-CT1; Figure S3: Full Blot x-CT2; Figure S4: Full Blot x-CT3; Figure S5: Full Blot x-CT4 and Figure S6: Full Blot Supplementary Figure S1 x-CT1.
