# Supplementary material for: Formyl-Peptide Receptor 2 Signaling Modulates SLC7A11/xCT Expression and Activity in Tumor Cells
Source: Antioxidants (Basel). 2024 Apr 30;13(5):552. doi: 10.3390/antiox13050552 (PMC11118824; doi:10.3390/antiox13050552)
Supplement: Supplementary file 1 [file antioxidants-13-00552-s001.zip › Full Blot revised_FC/S1 Supplementary Figure 1 xCT.pdf]

Supplementary Figure S1

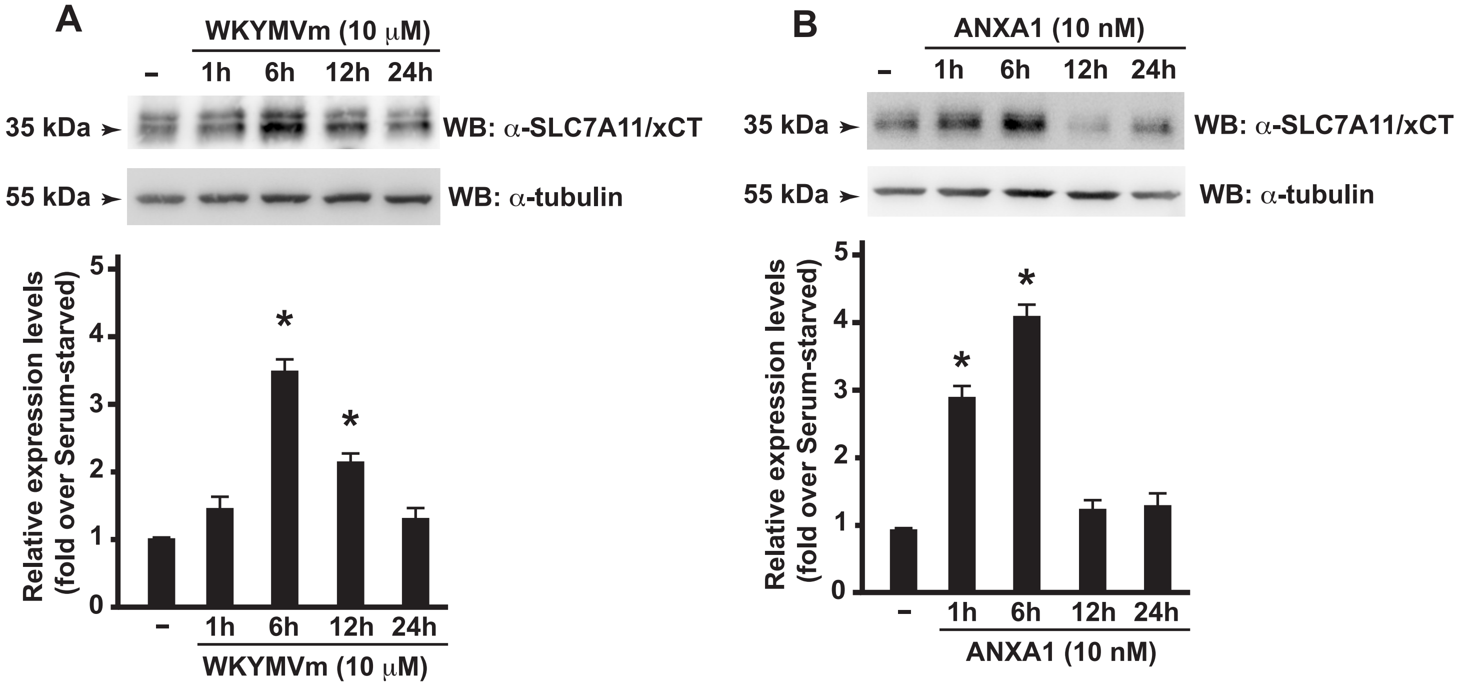

Supplementary figure1. HCT 116 cells were serum-starved for 24 hours and then stimulated for 1, 6, 12 or 24 hours with WKYMVm (A) or ANXA1 (B). Fifty-five micrograms of whole lysates were resolved on 10% SDS-PAGE and incubated with an anti-SLC7A11/xCT (α-SLC7A11/xCT) antibody. An anti-tubulin (α-tubulin) antibody was used as a control of protein loading. Data are representative of 3 different experiments. *p<0.05 com-pared to unstimulated cells.
