# Supplementary figures and images for: Formyl-Peptide Receptor 2 Signaling Modulates SLC7A11/xCT Expression and Activity in Tumor Cells
Source: Antioxidants (Basel). 2024 Apr 30;13(5):552. doi: 10.3390/antiox13050552 (PMC11118824; doi:10.3390/antiox13050552)

Supplementary Figure S6

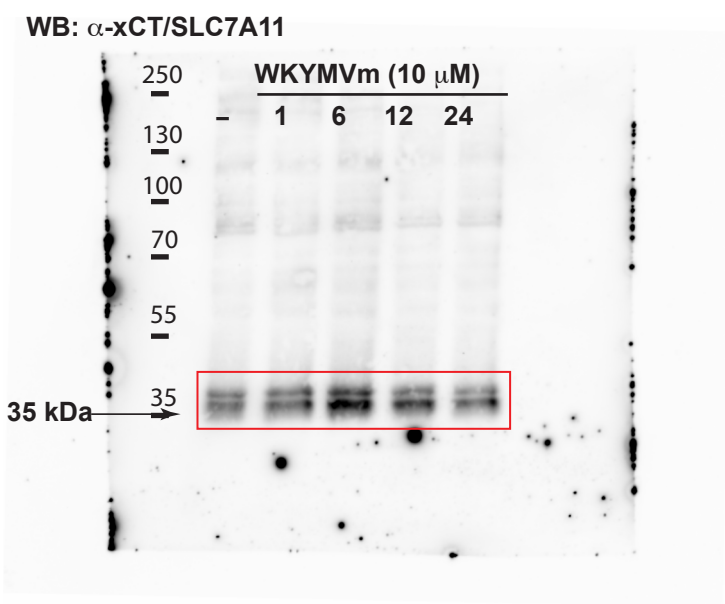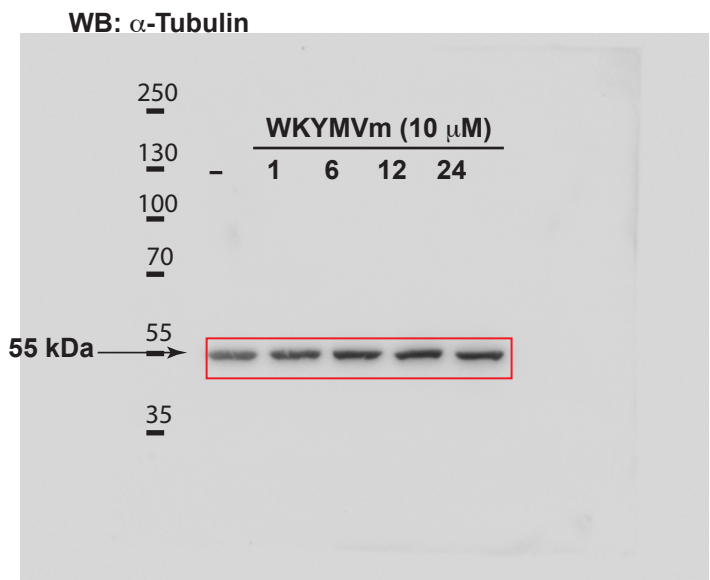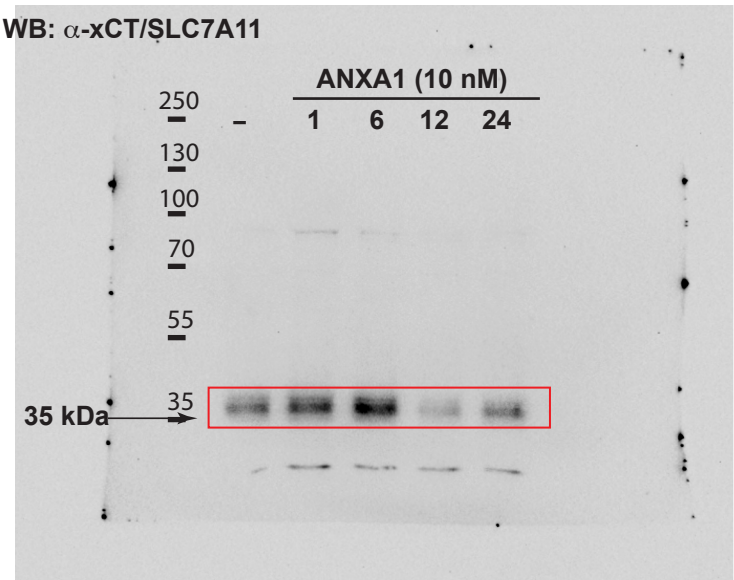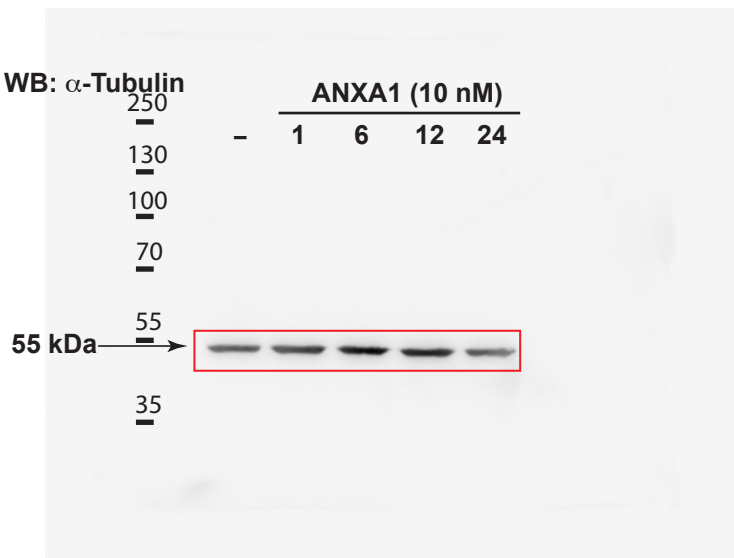

Supplement: Supplementary file 1 [file antioxidants-13-00552-s001.zip › Full Blot revised_FC/S6 Full Blot supplementary figure1 x-CT1.pdf]
